# Predictors of couple HIV counseling and testing among adult residents of Bukomero sub-county, Kiboga district, rural Uganda

**DOI:** 10.1186/s12889-015-2526-3

**Published:** 2015-11-24

**Authors:** Richard Muhindo, Annet Nakalega, Joyce Nankumbi

**Affiliations:** Department of Nursing, College of Health Sciences, Makerere University, P.O Box 7072, Kampala, Uganda; Mulago National Referral Hospital, Kampala, Uganda

**Keywords:** Predictors, Couple HIV Counseling and Testing, Rural Uganda

## Abstract

**Background:**

Studies have shown that couple HIV counseling and testing (CHCT) increased rates of sero-status disclosure and adoption of safer sexual behaviors with better linkage to treatment and care. However, current evidence suggests that new HIV infections are occurring among heterosexual couples in stable relationships where the majority of the individuals are not aware of their partner’s serostatus. This study examined the predictors of CHCT uptake among married or cohabiting couples of Bukomero sub-county Kiboga district in Uganda.

**Methods:**

This cross-sectional correlational study was conducted among 323 individuals who were either married or cohabiting, aged 18–49 years. Participants were enrolled from randomly selected households in Bukomero sub-county. Data were collected using an interviewer-administered questionnaire on socio-demographics, self-rating on awareness of CHCT benefits, couple discussion about HIV testing and CHCT practices. Couples were compared between those who had reported to have tested as a couple and those who had not. Binary logistic regression was performed to determine the adjusted odds ratio [aOR] and 95 % confidence intervals [CI] for CHCT uptake and the other independent variables.

**Results:**

Of the participants 288 (89.2 %) reported to have ever taken an HIV test only 99 (34.4 %) did so as a couple. The predictors of testing for HIV as a couple were discussing CHCT with the partner (adjusted odds ratio 4.95[aOR], 95 % confidence interval [CI]:1.99–12.98; *p* < 0.001), awareness of CHCT benefits (aOR 3.23; 95 % CI 1.78–5.87; *p* < 0.001) and having time to test as a couple (aOR 2.61; 95 % CI 1.22–5.61; *p* <0.05).

**Conclusion:**

Uptake of HIV counseling and testing among couples was low. Discussing CHCT with partner, awareness of CHCT benefits, and availability of time to test as a couple were predictive of CHCT uptake. Thus CHCT campaigns should emphasize communication and discussion of HIV counseling and testing among partners.

## Background

Uganda is one of two sub-Saharan African countries where the number of new HIV infection increased between 2005 and 2013 [1]. Of the 1.5,000 000 new HIV infections posted by the region in 2013, nearly 48 % were contributed by Uganda, South Africa and Nigeria [1]. Similar findings were reported in a national Ugandan survey where the proportion of the adult population living with HIV increased from 6.4 % in 2005 to 7.3 % in 2011 [2, 3]. Of the reported 130,000 new HIV infections in 2009 in Uganda, 43 % occurred in serodiscordant couples [4–7]. Studies evaluating the effectiveness of preventive interventions in discordant couples have shown that up to 80 % of new infections could be prevented by consistent condom use, 54 % from circumcision in negative male partners, 71 % from post exposure prophylaxis (PrEP), and 96 % from use of antiretroviral therapy (ART) by the HIV infected individuals [4, 8–15].

In efforts to reduce the number of new infections, Uganda has adopted a combination of HIV prevention approaches. The plan is to promote and scale up interventions both biomedical (ART, PrEP, male circumcision, and HIV counseling and testing) and behavioral change (condom use, and reduction in number of sexual partners), and also to address the structural drivers of HIV [16]. The current strategy highlights priority prevention interventions for particular categories of individuals, and specifically discordant couples with the importance of linking them into HIV care [16]. Current estimates indicate that 6 % of the couples in Uganda are discordant and that 43 % of the new infections that occurred in 2008 were among mutually monogamous heterosexual couples where many did not know their partner’s HIV status [3, 7]. Raising the levels of mutual HIV sero-status awareness among discordant couples could ultimately impact the proportion enrolled into care and prevention.

Couple HIV counseling and testing (CHCT) uptake is an important entry into HIV care and prevention [17–19]. CHCT is thus a key link to combination prevention interventions with ART, PrEP and male circumcision. CHCT is associated with partner disclosure and adoption of safer sexual practices [20–24]. When couples are tested together the burden of disclosure is reduced [25]. Nondisclosure of a positive sero-status was common among couples when either member went alone for testing [26, 27]. This is a drawback in the fight against HIV/AIDS especially when new infections are occurring in stable heterosexual relationships [27]. Knowledge of partner’s sero-status increased chances of being enrolled into HIV care and adoption of safe sex practices including condom use [28–30].

Emerging evidence indicates that up to three-quarters of new HIV infections could be prevented by couple HIV counseling and testing [31–34]. For concordant negative couples (both partners HIV negative), CHCT is important in the adoption of risk-reduction behaviors. It’s also important for concordant positive couples so that they can access available family planning, social services, treatment programs and safe child bearing. Further, CHCT is important to discordant couples, partners with different HIV status, to prevent HIV transmission to the uninfected partners, for social support and for treatment of the infected person. HIV testing is widely available in Uganda at stand alone voluntary counseling and testing (VCT) clinics, health centers and hospitals. However, despite the benefits associated with CHCT, few couples have mutually disclosed their HIV sero-status. Our study sought to determine the predictors of CHCT uptake among residents of Bukomero sub-county, Kiboga district. The district is located in the central part of the country where there is high discordance [3].

## Methods

### Study design and setting

A cross-sectional study was conducted in Bukomero sub-county between February and March, 2013. The sub-county is found in Kiboga a rural district in Central Uganda. The district has a population of 148,606 people with a third living in Bukomero [35].

### Study population and data collection procedures

The study participants included adult men or women aged 18–49 years who were either married or cohabiting for not less than 6 months at the time of data collection. Participants were recruited from randomly selected households in the four parishes of Bukomero. If the woman and man were both present in the household, they were interviewed as a couple. A structured self-report interview was used to collect data. The questionnaire was pretested on 20 adults who were married from Nangabo Sub-county in Wakiso district in January 2013. Adjustments were made to clarify some questionnaire items.

### Statistical analysis

In this study, testing for HIV as a couple, the primary outcome of interest was defined as whether a man or a woman in the current relationship had ever sought CHCT. It was measured on a dichotomous scale of “Yes” and “No”. The three major independent variables were the discussion of CHCT with the partner, perceived awareness of CHCT benefits, and willingness to go for CHCT. Discussing CHCT with the partner was defined as whether there were conversations about the need and benefits of CHCT. It was measured on a three point rank scale from very often to rare. Perceived awareness of CHCT benefits was defined by the participant’s self-evaluation of awareness of CHCT benefits. It was measured on a five point likert scale from strongly agree to strongly disagree. Participants were also asked to indicate whether they were or were not willing to go for CHCT.

Demographic data was collected on the duration of the relationship, nature of the co-habitation of the relationship (stay together under one roof or occasionally see each other), and having enough time as a couple to go for HIV counseling and testing. Others variables included religion, level of education and their perception of their participation in gainful work.

Statistical analysis included contingency tables and binary logistic regression. The three major independent variables were assessed using ordinal level of measurement and dichotomized during analysis. For perceived awareness of CHCT benefits, participants who rated themselves to be aware of CHCT benefits by selecting strongly agree or agree were considered to score high and those who selected strongly disagree, disagree or neutral were considered as unaware and were scored low. Contingency tables and correlation index procedures, association between outcome reference variable, testing for HIV as a couple and independent variables were examined. Variables that showed significant association in unavariable analysis (*p* < .05) with the outcome variable were further analyzed using binary logistic regression, a technique used to describe association between categorical outcome variable with categorical independent variables [36]. Individual odds and adjusted odds for the independent variables were determined. Analyses were performed using SPSS version 17.0.

### Ethical considerations

The study was approved by the Makerere University School of Health Sciences Institutional Review Board. All participants in the study provided written informed consent.

## Results

### Socio-demographic characteristics

The study enrolled 323 male and female participants who were married or cohabiting for a minimum of six months at the time of data collection. The median age of the participants was 25 years and the majority 240 (74 %) were females. High number of females was probably due to the time of day of the data collection when only the women were most likely to be home. Half the respondents 174 (54 %) had obtained a secondary education or higher, and 319 (99 %) were living as couples under one roof. The median duration of the current relationship was 4 years with 154 (48 %) reporting having been in relationship for more than 4 years. The majority 244 (75 %) reported discussing CHCT with their partner, 271 (84 %) reported willing to go for CHCT, and 129 (40 %) rated themselves low on awareness of CHCT benefits (see Table 1). Despite 253 (79 %) respondents reporting they had time as a couple to go for CHCT and 288 (89 %) had ever tested for HIV, only 99 (34 %) did so as a couple. The commonly cited reasons for participating in CHCT included: wanting to get married 99 (54 %), request during antenatal care 50 (27 %) and desire to know their HIV status 34 (19 %). Among the 99 respondents who tested as couples, 64 (65 %) had tested within the last year.

**Table 1:** Socio-demographic characteristics (*N* = 323)

Variables	Frequency (%)	Median	Interquartile range
Age		25 years	10 years
Gender			
Female	240 (74)		
Male	83 (26)		
Nature of relationship			
Staying together under one roof	319 (98.8)		
See each other occasionally	4 (1.2)		
Religion			
Christian	269 (8.3)		
Muslim	54 (16.7)		
Education level			
Primary or no formal education	149 (46)		
Secondary and above	174 (54)		
Duration in the relationship			
Less than 4 years	169 (52)	4 years	8 years
Greater than 4 years	154 (48)		
Ever tested for HIV			
Yes	288 (89)		
No	35 (11)		
Talking about CHCT in the relationship			
Very often	244 (76)		
Rarely	79 (24)		
Percieved awareness of the CHCT benefits			
High	194 (60)		
Low	129 (40)		
Last time tested asa couple			
within the last year	64 (65)		
within the last 2 years	20 (20)		
More than 3 years	15 (15)		

### Association between CHCT and independent variables

Bivariate analysis showed that there were significant statistical associations between discussed CHCT with partner (*p* < 0.001), perceived awareness of the CHCT benefits (*p* < 0.001), had time as couple for CHCT (*p* ≤ 0.001), were willing to go for CHCT (*p* < 0.009), duration of the relationship (*p* < 0.04) and participating in CHCT (see Table 2). Among the 99 participants who reported to have tested as a couple, 93 were identified as talking very often about CHCT within the relationship, 80 rated themselves high on self-perceived awareness of the benefits of CHCT, 89 reported having time as a couple for CHCT, 95 were willing to go for CHCT, and 62 were in the relationship of less than four years. Other variables such as education and religion showed no significant statistical association. Further, there was a significant statistical association between discussing CHCT with partner and perceived awareness of the CHCT benefits (*p* < .002). Likewise, 65 % of those who reported discussing CHCT with partner also rated themselves high on perceived awareness of the CHCT benefits, 82 % indicated having time as a couple for CHCT while 93.4 % were willing to go for CHCT.

**Table 2:** Associations between CHCT and independent variables (*N* = 288)

Variable	Yes	No	*P*-value
	(*N* = 99)	*N* (189)	
Religion			.250
Christians	87 (35 %)	159 (65 %)	
Muslim	12 (29 %)	30 (71 %)	
Level of education			.419
Primary or no formal education	46 (35 %)	84 (65 %)	
Secondary and above	53 (34 %)	105 (67 %)	
Duration in relationship			.04^*^
Less than 4 years	62 (39 %)	96 (61 %)	
Talking about CHCT in the relationship			.001^*^
Most often	93 (39.9)	140 (60.1)	
Rarely	6 (10.9)	49 (89.1)	
Perceived awareness of CHCT benefits			.001^*^
High awareness	80 (43.0)	106 (57.0)	
Low awareness	19 (18.6)	83 (81.4)	
Have time as couple for CHCT			.001^*^
Yes	89 (39.0)	139 (61.0)	
No	10 (16.7)	50 (83.3)	
Willingness to go for CHCT			.009^*^
Yes	95 (36.7)	164 (63.3)	
No	4 (13.8)	25 (86.2)	

*Statistically significant variables at *P* < 0.05

### Predictors of couple HIV counseling and testing (CHCT)

In the univariable analysis discussion of CHCT with their partner, awareness of the benefits of couple counseling and having time for CHCT were significantly associated with testing for HIV as a couple (see Table 3). The adjusted odds ratios for CHCT predictors were: discussion of CHCT with the partner (adjusted odds ratio 4.95[aOR]; 95 % confidence interval [CI]:1.99–12.98; *p* < 0.001), awareness of CHCT benefits (aOR 3.23; 95 % CI 1.78–5.87; *p* < 0.001) and had time to test as a couple (aOR 2.61; 95 % CI 1.22–5.61; *p* <0.05).

**Table 3:** Predictors of couple HIV counseling and testing; multivariate analysis

Variable	Odds Ratio (95 % CI)	*p*-value	Adjusted Odds ratio (95 % CI)	*p*-value
Duration in a relationship (< or > 4 years)	1.62 (.99–2.67)	.056	1.39 (.81–2.38)	.230
Discussing CHCT with partner	5.43 (2.23–13.18)	.001	4.95 (1.99–12.29)	.001
Awareness of CHCT benefits	3.28 (1.85–5.87)	.001	3.23 (1.78–5.87)	.001
Willingness to go for CHCT	3.62 (1.22–10.72)	.020	1.72 (.52–5.69)	.374
Having time as a couple for CHCT	3.20 (1.54–6.64)	.002	2.61 (1.22–5.61)	.014

## Discussion

This study focused on predictors of couple HIV counseling and testing (CHCT) of married or cohabiting men and women. The findings of the study show that 9 in 10 of the participants had ever taken an HIV test and 3 in 10 had tested for HIV as a couple. The predictors of CHCT were; discussion of CHCT with the partner, awareness of CHCT benefits and had time for CHCT.

In Uganda CHCT is highlighted as one of the key linkages to HIV care, treatment and prevention [37]. CHCT is particularly important because it reduces the burden of sharing one’s HIV positive status, provides a built-in support system which may aid in linking individuals with HIV to essential care and treatment services. This is important for discordant couples for which antiretroviral therapy may significantly reduce the risk of transmission [20, 38–40].

Our current findings, however, indicate that despite high (84 %) reported willingness to go for CHCT, uptake is still low. This demonstrates the intention-behavioral gap observed in a number of behavioral studies. For example some studies evaluating condom use have shown that up to 50 % of those who intend to use condoms never used them [41, 42]. Among the most frequent cited reasons for testing as a couple were; wanting to get married, meeting a request during antenatal care and desiring to know HIV status. In this study we found that couples who discussed CHCT with their partner were almost 5 times more likely to take an HIV test as a couple compared to those who did not. Discussing CHCT with partner could probably help couples reflects on the benefits of HIV testing, but also build their confidence and self-efficacy in confronting the challenges associated with disclosure and acceptance of results.

The findings of the study are similar to studies conducted in Nigeria, Mozambique and eastern Uganda in which low rates of couple HIV testing were reported [43–47]. While studies conducted in Zambia, Kenya and Tanzania demonstrated increased CHCT uptake when using a home-based approaches [48–53], this approach has not been scaled up in Uganda. Other studies have also established discussing CHCT with their partner, awareness of CHCT benefits and having time for CHCT as predictors of couple HIV testing. For example studies conducted in South Africa and Uganda established that sexual communication and prior CHCT discussion among partners was a strong predictor of taking an HIV test as a couple [43, 54]. Similarly, CHCT studies in Thailand, USA, Cameroon, Dominican Republic, Georgia and India among pregnant mothers and their partners have found similar results [55]. In a study on HIV self-testing in Nigeria awareness of CHCT benefits was associated with HIV testing [56]. Similar findings were identified in studies conducted in Northern Tanzania and South Africa [48, 57].

There are some study limitations related to the design and representativeness. The participants were recruited from Bukomera sub-county and as such may not be representative of the whole district. The study design was cross-sectional, and the findings could differ over a period of time. A longitudinal prospective study with a larger sample could provide a more complete understanding of the predictors of couples’ participation in CHCT.

However, our findings do support the model that partner communication and their awareness of CHCT benefits increases the chances of HIV counseling and testing among couples. Promoting couple communication on HIV testing should be key in CHCT promotional campaigns and programs.

## Conclusion

Uptake CHCT was low among heterosexual couples in a rural Ugandan setting and was significantly positively associated with discussion of CHCT with partner, awareness of CHCT benefits and reported availability of time. Our findings suggest that to increase HIV testing among couples, promotional campaigns should emphasize discussion of CHCT with partners.

## Abbreviations

AIC: Aids Information Center
AIDS: Acquired Immune Deficiency Syndrome
CHCT: Couple HIV Counseling and Testing
CVCT: Couple Voluntary Counseling and Testing
HCT: HIV Counseling and Testing
HIV: Human Immunodeficiency Virus
MOH: Ministry 0f Health
MSM: Men who have sex with Men
PMTCT: Prevention of Mother –to- Child Transmission
UAIS: Uganda Aids Indicator Survey
UNAIDS: The Joint United Nations Programme on HIV/AIDS
UNGASS: United Nations General Assembly Special Session on HIV/AIDS
VCT: Voluntary Counseling and Testing
VHCT: Voluntary HIV Counseling and Testing
WHO: World Health Organization.
